# Premature aging of leukocyte DNA methylation is associated with type 2 diabetes prevalence

**DOI:** 10.1186/s13148-015-0069-1

**Published:** 2015-03-28

**Authors:** Gidon Toperoff, Jeremy D Kark, Dvir Aran, Hisham Nassar, Wiessam Abu Ahmad, Ronit Sinnreich, Dima Azaiza, Benjamin Glaser, Asaf Hellman

**Affiliations:** Department of Developmental Biology and Cancer Research, Institute for Medical Research Israel-Canada, Hebrew University-Hadassah Medical School, Jerusalem, 91120 Israel; Epidemiology Unit, Braun School of Public Health and Community Medicine, Hebrew University and Hadassah Medical Organization, Jerusalem, Israel; Cardiology Department, Hadassah-Hebrew University Medical Center, Hadassah Medical Organization, Ein Kerem, Jerusalem, Israel; St Joseph Hospital (East Jerusalem), Jerusalem, Israel; School of Computer Science and Engineering, Hebrew University, Jerusalem, Israel; Endocrinology and Metabolism Service, Department of Internal Medicine, Hadassah-Hebrew University Medical Center, Hadassah Medical Organization, Ein Kerem, Jerusalem, Israel

**Keywords:** Type 2 diabetes, DNA methylation, Epigenetic aging, Leukocytes, East Jerusalem Palestinians, Ashkenazi Jews, Population epigenetics, Ethnic groups

## Abstract

**Background:**

Type 2 diabetes mellitus (T2D) is highly prevalent in Middle-Eastern and North African Arab populations, but the molecular basis for this susceptibility is unknown. Altered DNA methylation levels were reported in insulin-secreting and responding tissues, but whether methylation in accessible tissues such as peripheral blood is associated with T2D risk remains an open question. Age-related alteration of DNA methylation level was reported in certain methylation sites, but no association with T2D has been shown. Here we report on a population-based study of 929 men and women representing the East Jerusalem Palestinian (EJP) Arab population and compare with the findings among Israeli Ashkenazi Jews. This is the first reported epigenetic study of an Arab population with a characteristic high prevalence of T2D.

**Results:**

We found that DNA methylation of a prespecified regulatory site in peripheral blood leukocytes (PBLs) is associated with impaired glucose metabolism and T2D independent of sex, body mass index, and white blood cell composition. This CpG site (Chr16: 53,809,231-2; hg19) is located in a region within an intron of the *FTO* gene, suspected to serve as a tissue-specific enhancer. The association between PBL hypomethylation and T2D varied by age, revealing differential patterns of methylation aging in healthy and diabetic individuals and between ethnic groups: T2D patients displayed prematurely low methylation levels, and this hypomethylation was greater and occurred earlier in life among Palestinian Arabs than Ashkenazi Jews.

**Conclusions:**

Our study suggests that premature DNA methylation aging is associated with increased risk of T2D. These findings should stimulate the search for more such sites and may pave the way to improved T2D risk prediction within and between human populations.

**Electronic supplementary material:**

The online version of this article (doi:10.1186/s13148-015-0069-1) contains supplementary material, which is available to authorized users.

## Background

The prevalence of type 2 diabetes mellitus (T2D) is particularly high in Arab populations; six of the top ten ranked countries by diabetes prevalence are Arab Middle-Eastern countries, the prevalence in Middle-Eastern and North African countries being 64% higher than in European countries [1-5]. Among Arabs in Israel, the prevalence of T2D was reported to be almost twofold higher and the age of onset 11 years earlier than among Jews of same geographic area and age (average onset at age 57 *vs*. 68 years in Arabs and Jews, respectively) [6]. The molecular basis for this higher prevalence and earlier age of onset is unknown.

Altered DNA methylation levels were reported in insulin-secreting and insulin-responding tissues of T2D patients [7-10], but whether methylation in peripheral blood leukocytes (PBLs) is associated with T2D risk remains an open question. We previously reported a genome-wide screen of DNA methylation differences among T2D cases and controls in the Ashkenazi Jew (AsJ) population. This methylation site in the first intron of the *FTO* gene showed, in the context of a longitudinal case–control design nested in a cohort, evidence for T2D-related hypomethylation in PBLs of healthy young men and women years before the onset of the disease, independently of the effects of risk sequence alleles [11].

Further investigation suggested some clues as to the possible function of this methylation site. First, it is located only 16 bases from SNP rs1121980 that was repeatedly found to be associated with T2D and obesity [12-14]. Second, based on its intermediate methylation level and histone modification marks, this site may be linked to regulation of gene expression since it resides within a putative tissue-specific enhancer. Moreover, it resides within a perfect binding motif of *USF1*/*2* transcription activators that play an important role in the regulation of glucose and lipid metabolism in response to insulin, as well as participating in beta-cell development [15,16]. Third, in spite of the established association between *FTO* alleles and obesity, our analysis indicates no association between methylation at this site and body mass [11]. These results are in line with the reported residual association of SNPs in the region with T2D independent of obesity [17,18]. Finally, Smemo *et al*. recently reported evidence suggesting that this region acts as an enhancer in the human brain and that it may directly interact with and control the expression of the IRX3 promoter, which in turn regulates body mass and composition [19].

Here we investigate a population-based sample drawn from the East Jerusalem Palestinian (EJP) Arab population. The results reveal a significant association between PBL DNA hypomethylation and T2D that varied by age, suggesting differential patterns of methylation aging in healthy and diabetic individuals. This is the first reported epigenetic marker to be associated with T2D in any high T2D prevalence Arab population.

## Results

Characteristics of the EJP study sample are shown in Table 1. The Palestinian sample comprised 929 individuals (53% men) of mean age 51.8 years (age range at examination 25.7 to 77.4 years). In accordance with previous reports of high T2D prevalence in Arab populations [1-6], 28.7% of the EJP participants were fully diabetic, and a further 20.9% had impaired glucose metabolism (IGM). Age was, as expected, strongly associated with diabetes status, increasing monotonically from a mean of 44.1 years in the normal GM group to 60.3 years in the T2D group. Body mass index (BMI) increased monotonically across the four diabetes categories from 27.8 kg/m^2^ in the normal GM group to 31.4 kg/m^2^ in the T2D group.

**Table 1:** **Characteristics of the EJP and Ashkenazi Jewish study participants**

**Study design and sample**	**Diabetes status**	**Number (** ***n*** **)**	**% of total**	**% of men**	**Mean Age (years)**	**Mean BMI (kg/m** ^**2**^ **)**	**Mean methylation (%)**	**SD of methylation**
PS-EJP	Group 1: normal GM	295	31.8	51.2	44.1	27.8	31.3	8.5
PS-EJP	Group 2: borderline GM	173	18.6	59.5	48.0	29.4	31.8	9.0
PS-EJP	Group 3: IGM	194	20.9	50.5	55.2	30.9	29.5	8.7
PS-EJP	Group 4: T2D	267	28.7	52.8	60.3	31.4	28.1	8.4
PS-EJP	Groups 1 + 2	468	50.4	54.3	45.5	28.4	31.5	8.7
PS-EJP	Groups 3 + 4	461	49.6	51.8	58.1	31.2	28.7	8.6
CC-AsJ	No diabetes	348	55.3	41.1	58.7	22.5	30.8	8.6
CC-AsJ	T2D	281	44.7	47.7	64.5	29.0	28.1	7.5

EJP: East Jerusalem Palestinian; PS: Cross-sectional population-based sample; GM: Glucose metabolism; IGM: Impaired glucose metabolism; T2D: Type 2 diabetes. AsJ: Ashkenazi Jewish; CC: Cross-sectional case–control.

### Association of methylation with T2D prevalence in the EJP sample

Multiple factors may influence methylation level. We assessed the methylation association with T2D taking account of potential confounders and effect modifiers including age, sex, BMI, and white blood cell composition (lymphocyte to granulocyte ratio).

We examined these potential effects using two different definitions of the disease status: first, we defined the outcome as T2D categories 3 and 4 (IGM + T2D, *n* = 461) *vs*. categories 1 and 2 (normal GM + borderline GM, *n* = 468), and second, we restricted the comparison to category 4 (T2D patients, *n* = 267) *vs*. category 1 (normal GM subjects, *n* = 295). For each of the two comparisons, we constructed three models: model 1 included methylation of the *FTO* site and age adjustment using linear and quadratic terms for age; model 2 tested for interaction and included the model 1 variables in addition to a multiplicative term of linear age and methylation; and model 3 included additional adjustment for BMI, sex, and the lymphocyte to granulocyte ratio (Table 2). Differences in PBL methylation could result from altered methylation of lymphocytes or granulocytes (or both), or from differing proportions of cell types with fixed methylation [11]. We introduced the lymphocyte/granulocyte ratio in the regression models to account for the possibility that methylation differences are not in the actual methylation levels but rather a result of differences in white blood cell composition.

**Table 2:** **Association of PBL methylation with T2D status in EJP - multivariable logistic modeling**

**Model**	**Dependent variable**	**Number (cases, non-cases)**	**Main effect odds ratio**	***P*** **-value**	**Interaction odds ratio**	***P*** **-value**
1	T2D + IGM (groups 1 + 2 *vs.* groups 3 + 4)	929 (461, 468)	0.979	0.014	NA	NA
2			0.894	0.006	1.002	0.023
3			0.909	0.021	1.001	0.045
1	T2D (group 1 *vs.* group 4)	562 (267, 295)	0.982	0.142	NA	NA
2			0.846	0.011	1.003	0.021
3			0.853	0.02	1.003	0.022

Model 1: Independent variables: continuous age, age^2^, methylation. Model 2: Independent variables: continuous age, age^2^, methylation, methylation × age interaction. Model 3: Independent variables: continuous age, age^2^, methylation, methylation × age interaction, BMI, sex, lymphocyte to granulocyte ratio. Group 1: Normal glucose metabolism (GM); Group 2: Borderline GM; Group 3: Impaired GM; Group 4: T2D; NA: Not applicable (*i.e.* no interaction term was introduced).

In the comparison of the combined group of T2D and IGM subjects *vs*. the normal and the borderline GM subjects, methylation was significantly inversely associated with diabetes and IGM in the age adjusted model 1 (odds ratio (OR) = 0.979, *P* = 0.014). Introduction of the interaction term of age and methylation (model 2) further improved the fit. The main effect for methylation (OR = 0.894, *P* = 0.006) indicates that for a 1 unit increase in methylation, the odds of T2D and IGM decrease by a factor of 0.894, whereas the interaction term (OR = 1.002, *P* = 0.023) points to an attenuation of the inverse association with increasing age, such that for each 1 year increase in age, the odds ratio increases by a factor of 1.002 toward the null value (and beyond). Adjustment for the additional covariates (model 3) modestly attenuated the association (main effect: OR = 0.909, *P* = 0.021; interaction term: OR = 1.001, *P* = 0.045).

In the T2D *vs*. normal GM comparison, methylation was inversely but not significantly associated with T2D (OR = 0.982, *P* = 0.142) in model 1. Introduction of the interaction term (model 2) significantly improved the fit of the model (main effect for methylation: OR = 0.846, *P* = 0.011; interaction term: OR = 1.003, *P* = 0.021). Addition of the possible confounding variables (model 3) had little effect (main effect for methylation: OR = 0.853, *P* = 0.020; interaction term: OR = 1.003, *P* = 0.022). The weakening of the inverse association of methylation with T2D with advancing age in this restricted comparison tended to be larger than in the full sample.

In line with our previous report of no significant association between methylation level and BMI among AsJs or in the Jerusalem LRC cohort [11], the analysis of the EJP sample revealed no association between BMI and methylation level (Additional file 1: Figure S1).

Taken together, these data indicate an inverse association of PBL methylation level with T2D that is independent of sex, BMI, and the lymphocyte to granulocyte ratio, but weakens with increasing age.

### Methylation by age within T2D categories

**Figure 1:**
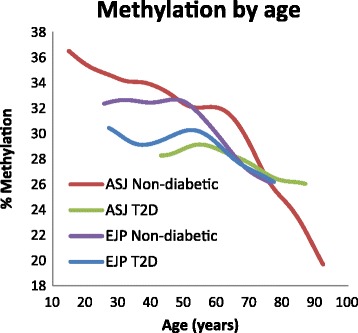
**Methylation** ***vs.*** **age in T2D and T2D-free individuals from the Ashkenazi Jewish and the EJP samples.**

We analyzed the cross-sectional association of methylation status by age within T2D categories. The curvilinear age association appeared to differ between diabetic and non-diabetic subjects: in the combined group of normal and borderline GM EJPs, the methylation level was relatively high up to age 50 and then sharply decreased (Figure 1). T2D and IGM subjects, in contrast, were already hypomethylated at young ages. The methylation status of the normal group intersected with the diabetic group between ages 55 and 65. Thus, the methylation differences that were apparent between the young diabetic subjects and the normal GM group no longer existed at the older ages. Of interest, the average age of T2D onset reported for Arabs in Israel (57 years) [6] is close to the age of the shift toward demethylation and corresponds with the age of intersection between the methylation levels of normal and diabetic subjects.

### Comparison with an Ashkenazi Jewish sample

We compared the association of methylation with T2D status in the Israeli Jewish and Palestinian Arab populations. We have previously reported that non-diabetic AsJs had higher methylation levels than diabetic patients between ages 40 and 70 [11]. Here we analyzed the methylation levels of 281 AsJ T2D patients aged 40 to 87 and 348 non-diabetic AsJ controls aged 10 to 92. The methylation status of the diabetic and the non-diabetic groups across the age range was very similar to that observed in the EJP population sample (Table 1).

As in the EJPs, the methylation levels of non-diabetic AsJs were relatively high at young ages and decreased with age, particularly after age 65. In contrast, diabetic AsJs were already hypomethylated at the young ages. The methylation status of the normal and diabetic AsJ groups intersected around age 73, and thus the methylation differences between diabetic and non-diabetic subjects observed at the younger ages were no longer evident in the older ages (Figure 1). As in the EJP population, the average age of T2D onset (68 years among Israeli Jews [6]) is just above the age of the shift toward accelerated hypomethylation (65 years) and corresponds with the age at which the methylation level of normal subjects intersects with the methylation level of T2D patients.

Across most of the age distribution, non-diabetic EJPs were hypomethylated compared to their AsJ counterparts, suggestive of a mechanistic link between the earlier hypomethylation of the EJPs and the higher T2D prevalence typical of this population. Furthermore, accelerated demethylation and the intersection with the methylation level of T2D subjects occur about 10 years earlier in the EJPs as compared to the AsJs, corresponding with a similar earlier onset of T2D in this population.

## Discussion

In this study, we demonstrated and confirmed the occurrence of T2D-related DNA methylation differences in PBLs. Despite methodological differences between the studies, the main findings of the original study in Israeli Jews were replicated in a sample of urban Palestinian Arabs. These findings include large between-individual variation in methylation, modest but consistent hypomethylation of T2D *vs*. T2D-free individuals, similar effect sizes, a similar dependence of this association on age (such as attenuation of the association in the older age groups), and independence of T2D-related methylation from the effects of BMI, sex, and white blood cell composition.

We have applied accepted T2D classification methods and tested methylation differences between various disease groups. The results indicate significant differences independent of the classification method (Table 2).

Despite sharing similarities in their genetic backgrounds [20,21], Palestinian Arab and Israeli Jewish populations show extensive phenotypic differences including health behaviors [22,6], cardiometabolic characteristics including considerable differences in T2D occurrence [23,6] and HDL-cholesterol concentrations [23], coronary heart disease incidence [24] and mortality [25], and life expectancy [26]. Considering these diverse phenotypes and the different lifestyles of the populations from which the samples were drawn, it is striking that *FTO* methylation is associated with T2D in both populations. These findings suggest that hypomethylation of the *FTO* site is a common marker of diabetes in PBLs rather than a population-specific phenomenon, though examination of more genetically diverse populations remains to be done.

A growing body of evidence indicates frequent associations between DNA methylation levels of PBL and other tissues and organs [27]. Disease-related methylation in PBLs might be due to the widespread influence of polymorphic sequences on the methylation levels of nearby sites [28-30]. Hence, if the alleles of a given disease-associated polymorphic sequence differentially affect methylation levels, they are expected to create a disease-associated methylation pattern. Such sequence-influenced methylation sites are common in the human genome [29] and were previously associated with T2D in PBLs [31]. However, extensive sequencing of DNA molecules containing both T2D-related methylation and sequence variations revealed that the methylation site we have analyzed is independently associated with diabetes [11]. Although *cis* effects of very distant sequences or effects in trans cannot be ruled out, *cis*-sequence-influenced methylation is unlikely to account for the observed T2D-related methylation in blood. Alternative mechanisms might include early development patterning of inter-individual differences shared by disease-related and unrelated tissues, or blood-based mechanisms. We have tested and rejected the possibility of differential white blood cell composition as a mechanism underlying diabetes-related methylation. However, other blood-based mechanisms are possible, including for example differential activity states of circulating PBLs between diabetic and non-diabetic individuals. Further examination of the PBL-differentiating site in T2D-related tissues may indicate which one of the above possibilities holds.

The molecular basis underlying the large diversity in the prevalence of T2D among human populations is currently unclear, but genetic and environmental factors are almost certainly both involved. The molecular basis for the higher prevalence and earlier age of onset of T2D in Arab populations is unknown. We observed similar trends of decreasing methylation with age in Palestinian Arabs and Israeli Jews, but the Palestinians were hypomethylated compared to Jews at all ages evaluated and attained the average methylation level of T2D individuals about 10 years earlier than Jews (Figure 1). The interpretation of this difference is complex. We suspect that the hypomethylation of the normal EJP population sample is not attributable solely to genetic differences between the populations but rather involves environmental differences such as diet and lifestyle, possibly as early as the intrauterine period. In turn, these methylation differences may define, or be associated with, altered gene expression profiles. Given that hypomethylation in PBLs distinguished young healthy individuals who later become diabetic from those who remained healthy during a 13-year follow-up [11], the lower methylation levels of the EJPs might predispose them to higher T2D risk.

## Conclusions

This is the first reported epigenetic marker to be associated with T2D in an Arab population. DNA hypomethylation in PBLs, which is greater among Arabs than Ashkenazi Jews, signifies increased T2D risk. The association between PBL hypomethylation and T2D varied by age, suggesting differential patterns of methylation aging in healthy and diabetic individuals and between ethnic groups. We expect that more PBL methylation sites in the genome may be additively associated with T2D risk. These findings may lead to a better understanding of the mechanisms underlying T2D.

## Methods

### Study design

The EJP study is a population-based, cross-sectional study. We examined the DNA methylation of a single CpG dinucleotide in DNA from peripheral white blood cells in a sample of 929 Palestinians. This specific CpG in an intron of the *FTO* gene (Chr16: 53,809,231-2; hg19), which is not covered by any of the commercially available DNA methylation analysis tools, was examined by an assay we customized in our lab as described below. The association between methylation levels and individual phenotypes, specifically T2D, was analyzed and compared to similar analyses performed in a separate case–control study of 629 Israelis of Ashkenazi Jewish descent.

### Subjects and population cohorts

1. The EJP sample of the Jerusalem Palestinian-Israeli Risk Factor Study was described in detail elsewhere [32]. Briefly, an age-sex-stratified random sample of 2,000 Palestinian Arabs aged 25 to 77, residents of East Jerusalem, was drawn from the Israel national population registry (in which EJPs with the legal status of permanent residents of Israel are recorded). Of these 70.5% could be located. After exclusion of those not meeting the eligibility criteria (10.4%), 970 EJPs were examined (response rate of 76.7% among those located), and 929 were successfully analyzed for methylation levels. Participants were recruited between 2005 and 2008 at the St. Josef Hospital in East Jerusalem for face-to-face interviews and clinical measurements performed by trained personnel. The interview included sociodemographic characteristics and information about health behaviors and health status. All subjects provided signed informed consent. The study was approved by the St. Josef Hospital and Hadassah Medical Center Ethics (Helsinki) Committees and the Israel Ministry of Health National Review Board for Genetic Studies.
2. Participants in the cross-sectional case–control study were Israeli residents of Jewish Ashkenazi origin (four grandparents): T2D subjects (*n* = 281) were ascertained by the Israel Diabetes Research Group, were treated for T2D, and were at least 10 years post-diagnosed at the time of DNA collection, as previously described [11]. The control group (*n* = 348) comprised non-diabetic subjects with parents free of diabetes that were drawn from three sources: the Israel Diabetes Research Group (*n* = 188), the Jerusalem Perinatal Study [33] (*n* = 96), and people undergoing health screening examinations at a hospital-based clinic (*n* = 64). In contrast with a previous report that included controls aged 40 to 70 years from these same sources [11], here the age was unrestricted and included subjects aged 11 to 92 years. DNA samples obtained from peripheral white blood cells were analyzed anonymously. All subjects provided signed informed consent. The study was approved by the Hadassah Medical Center Helsinki Review Committee, the Shaare Zedek Medical Center Helsinki Review Committee, and the Israeli National Helsinki Committee for Genetic Studies.

### Classification of T2D status

Diabetes classification was based on combinations of a reported prior physician’s diagnosis, medication for diabetes, and fasting and 2 h post-challenge glucose levels as follows:

- Group 1. Normal glucose metabolism (NGM): (fasting glucose (FG) < 100 mg/dl) *and* (2-h post 75 g oral glucose challenge (2HG) < 140 mg/dl) *and* (no diabetes diagnosis or treatment)
- Group 2. Borderline glucose metabolism (BGM): (99 < FG < 110 mg/dl) *and* (2HG < 140 mg/dl) *and* (no diabetes diagnosis or treatment)
- Group 3. Impaired glucose metabolism (IGM): (109 < FG < 126 mg/dl) *or* (140 ≤ 2HG <200 mg/dl) *or* (group 2 with a reported diabetes diagnosis but no treatment)
- Group 4. Diabetes: (FG > 125 mg/dl) *or* (2HG > 199 mg/dl) *or* (diabetes treatment).

The AsJ study was a case–control study in which patients previously diagnosed with T2D were recruited from diabetes outpatient clinics and a control group of diabetes-free individuals was assembled as described above. T2D diagnosis by a physician was verified before inclusion in the study.

### DNA methylation analysis

DNA samples were extracted from PBLs, treated with bisulfite (EZ-DNA kit, Zymo Research, Irvine, CA, USA) and polymerase chain reaction (PCR) amplified using the forward primer 5′GATAGGTAGGTAGGTGGATTTGAAATT and the reverse biotinylated primer 5′biotin-ACAACAACTAACTTAATAAACCCTCAAT. The PCR products were purified, quantified, and sequenced on a PyroMark Q24 bench-top device (Qiagen, Venlo, Limburg, the Netherlands) from the internal primer 5′GGTGGATTTGAAATTTTATATAGTA. All samples were coded and analyzed anonymously and blinded as to their diabetes status through the DNA purification, amplification, and methylation analysis steps.

### Quality control

Analyses were performed in 96-well plates. Within- and between-assay (plate) repeatability was assessed by replication of randomly selected samples as follows: Each 96-well plate was assigned 90 study samples and 6 technical replicates. Of these replicates, half were duplicates from the study samples in the same 96-well plate and the other half were duplicates from those in a separate plate. Thus, replication included all analysis steps; half of the replicates were included in the original plates (‘within’) and half were assigned to other plates (‘between’). The within-assay and between-assay coefficients of variation were 5.0% and 7.8%, respectively. The within- and between-assay intraclass correlation coefficients were 0.973 (95% CI 0.943 to 0.988) and 0.889 (95% CI 0.787 to 0.944), respectively (Additional file 2: Figure S2).

### Statistical analyses

Logistic regression with diabetes status as the dependent variable was used to adjust methylation levels for linear and quadratic terms of age and subsequently also for BMI, sex, and white blood cell composition. The effect modification of the methylation-T2D association was evaluated by introduction of a multiplication term of linear age and methylation. The contribution of the interaction term was assessed by the difference in the log likelihood before and after introduction of the term. All analyses presented satisfied the Hosmer-Lemeshow test for goodness of fit.

## Additional files

Additional file 1: Figure S1. Scatter plots showing the correlation between methylation and BMI among EJP. **A**. All participants. **B**. T2D-free participants from groups 1 and 2. **C**. IGM and T2D participants from groups 3 and 4. Additional file 2: Figure S2. Within and between plate reproducibility of DNA methylation pyrosequencing. Samples were allocated randomly to 96-well plates for bisulfite conversion, amplification and pyrosequencing with technical replicates in each plate. Each 96-well plate contained three within-plate replicates and three replicates from other 96-well plates. The within-plate replicates (A) and between-plates replicates (B) correlation coefficients (*r*) and coefficients of variation (CV) are shown.

## Abbreviations

AsJ: Ashkenazi Jewish
CC: cross-sectional case–control
EJP: East Jerusalem Palestinian
GM: glucose metabolism
IGM: impaired glucose metabolism
PS: cross-sectional population-based sample
T2D: type 2 diabetes
