# Supplementary material for: Premature aging of leukocyte DNA methylation is associated with type 2 diabetes prevalence
Source: Clin Epigenetics. 2015 Mar 28;7(1):35. doi: 10.1186/s13148-015-0069-1 (PMC4379765; doi:10.1186/s13148-015-0069-1)
Supplement: Additional file 1: Figure S1. — Scatter plots showing the correlation between methylation and BMI among EJP. A. All participants. B. T2D-free participants from groups 1 and 2. C. IGM and T2D participants from groups 3 and 4. [file 13148_2015_69_MOESM1_ESM.pptx]

## Slide 1
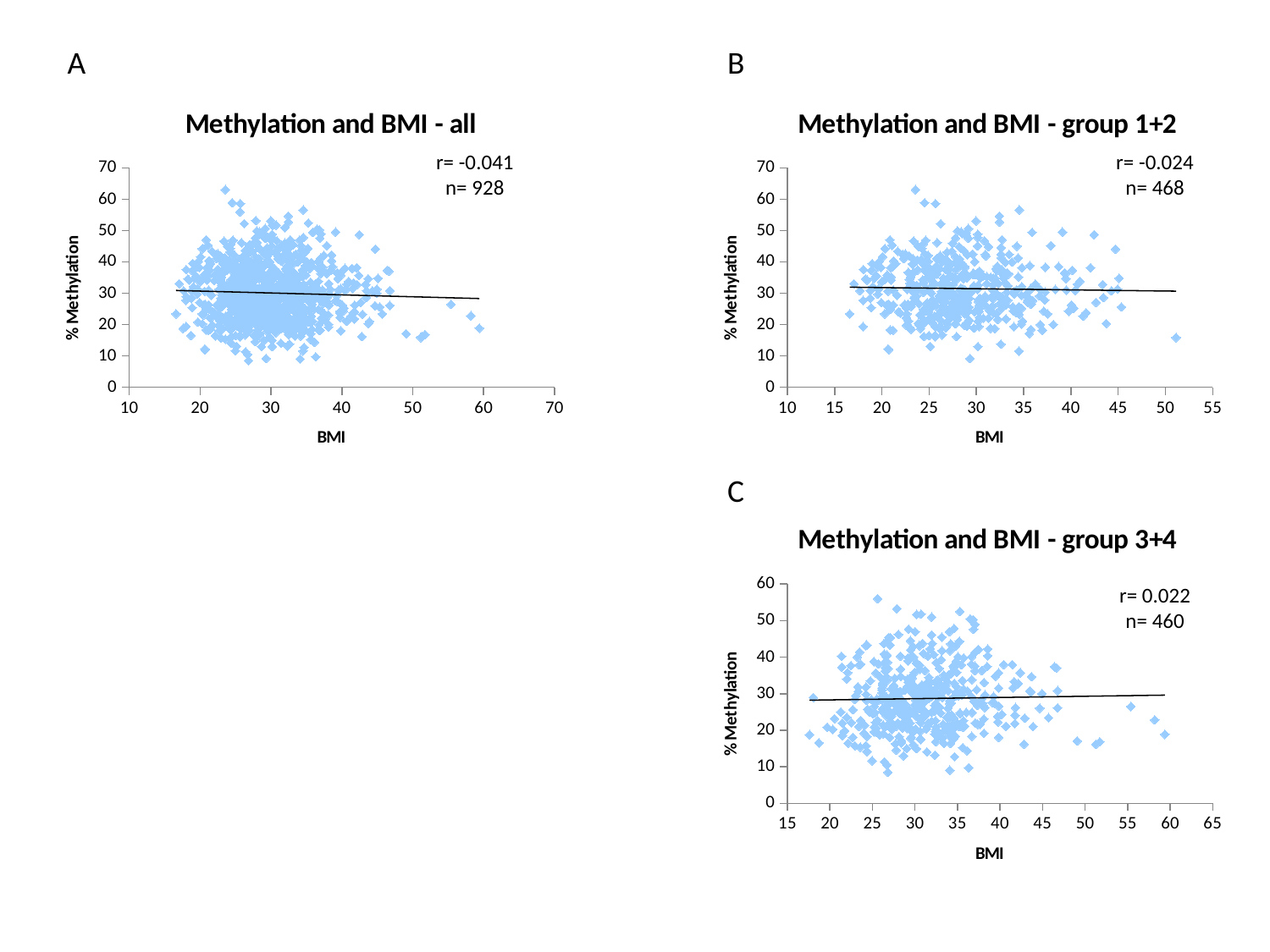

A
B
### Chart: Methylation and BMI - all
| Category | |
|---|---|
### Chart: Methylation and BMI - group 1+2
| Category | |
|---|---|r= -0.041
n= 928
r= -0.024
n= 468
C
### Chart: Methylation and BMI - group 3+4
| Category | |
|---|---|r= 0.022
n= 460
