# Supplementary material for: Premature aging of leukocyte DNA methylation is associated with type 2 diabetes prevalence
Source: Clin Epigenetics. 2015 Mar 28;7(1):35. doi: 10.1186/s13148-015-0069-1 (PMC4379765; doi:10.1186/s13148-015-0069-1)
Supplement: Additional file 2: Figure S2. — Within and between plate reproducibility of DNA methylation pyrosequencing. Samples were allocated randomly to 96-well plates for bisulfite conversion, amplification and pyrosequencing with technical replicates in each plate. Each 96-well plate contained three within-plate replicates and three replicates from other 96-well plates. The within-plate replicates (A) and between-plates replicates (B) correlation coefficients (r) and coefficients of variation (CV) are shown. [file 13148_2015_69_MOESM2_ESM.pptx]

## Slide 1
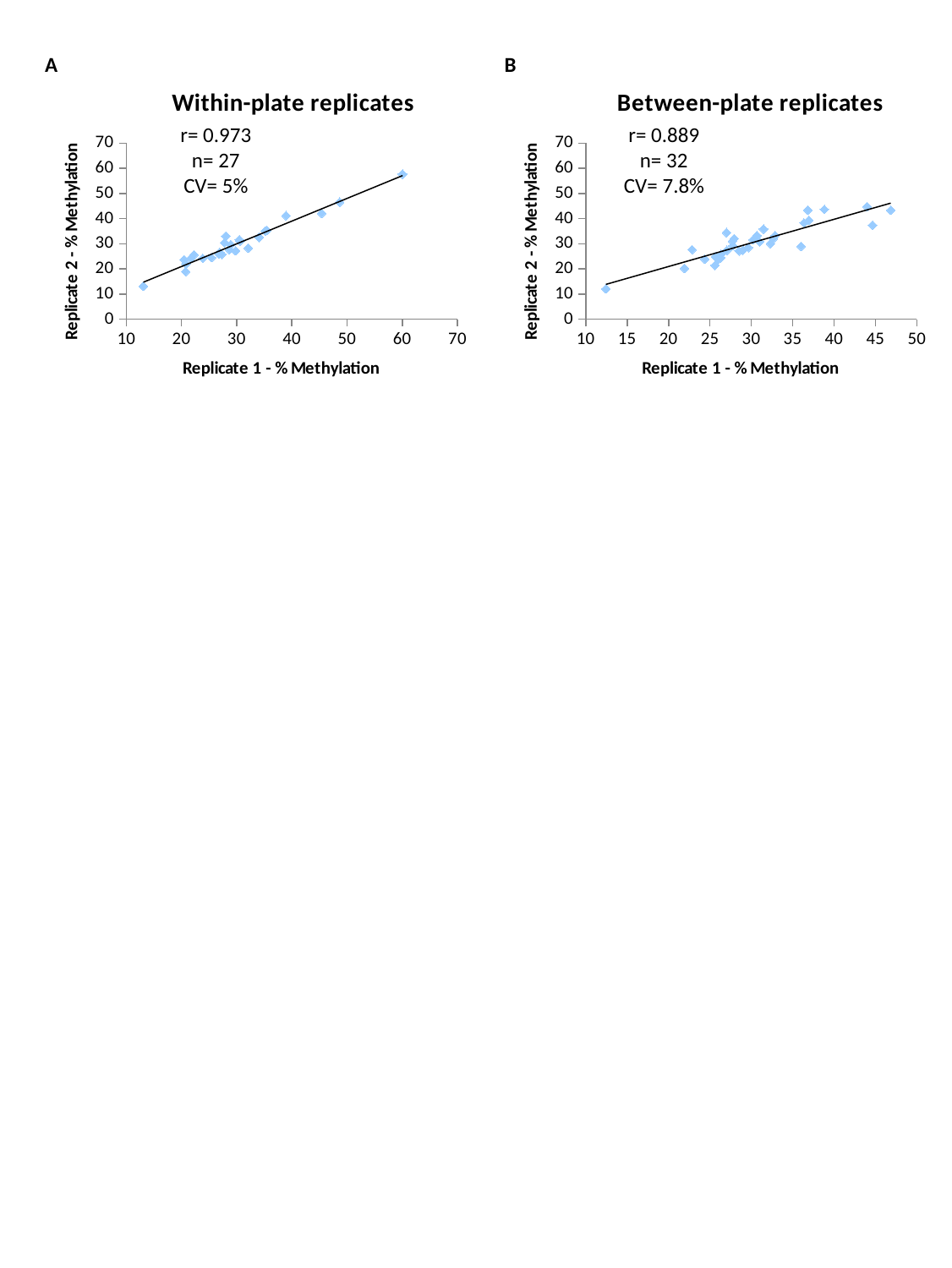

A
B
### Chart: Within-plate replicates
| Category | |
|---|---|
### Chart: Between-plate replicates
| Category | |
|---|---|
### Chart
| Category |
|---|r= 0.973
n= 27
CV= 5%
r= 0.889
n= 32
CV= 7.8%
